# Injury characteristics in Norwegian male professional football: A comparison between a regular season and a season in the pandemic

**DOI:** 10.3389/fspor.2022.915581

**Published:** 2022-10-21

**Authors:** Torstein Dalen-Lorentsen, Thor Einar Andersen, Christian Thorbjørnsen, Michael Brown, David Tovi, Anders Braastad, Tom Gerald Lindinger, Christian Williams, Eirik Moen, Benjamin Clarsen, John Bjørneboe

**Affiliations:** ^1^Department of Sports Medicine, Oslo Sports Trauma Research Center, Norwegian School of Sports Sciences, Oslo, Norway; ^2^Rosenborg Ballklubb, Trondheim, Norway; ^3^Bodø Glimt FK, Bodø, Norway; ^4^Brann Sportsklubb, Bergen, Norway; ^5^Odds Ballklubb, Skien, Norway; ^6^Department of Physiotherapy, Faculty of Health Sciences, Oslo Metropolitan University, Oslo, Norway; ^7^Kristiansund Ballklubb, Kristiansund, Norway; ^8^Stabæk Fotball, Bekkestua, Norway; ^9^Centre for Disease Burden, Norwegian Institute of Public Health, Bergen, Norway; ^10^Department of Physical Medicine and Rehabilitation, Oslo University Hospital, Oslo, Norway

**Keywords:** injuries, sports medicine, epidemiology, match congestion, soccer (football)

## Abstract

The Coronavirus Disease-19 (COVID-19) pandemic forced the Norwegian male premier league football season to reschedule, reducing the fixture calendar substantially. Previous research has shown that a congested match schedule can affect injury rates in professional football. Therefore, we aimed to investigate whether the Norwegian premier league teams suffered more injuries in the more match congested 2020 season than in the regular 2019-season. We invited all teams having participated in both seasons to export their injury data. Only teams that used the same medical staff to register injuries in both seasons were included, and to maximize data comparability between seasons, we applied a time-loss injury definition only. Seven of 13 teams agreed to participate and exported their injury data. Both seasons had 30 game weeks, but the 2020 season was 57 days shorter than the 2019 season. The match injury incidence did not differ significantly [incidence rate ratio 0.76 (0.48–1.20; *p* = 0.24) in the 2020 season compared to the 2019 season. Furthermore, we found no differences in the number of injuries, days lost to injury, matches missed to injury, or injury severity. We could not detect any differences between the two seasons, suggesting the congested match calendar combined with the safety measures in the 2020 season can be a safe alternative in future seasons.

## Introduction

Following the world-wide spreading of the Severe Acute Respiratory Syndrome Coronavirus 2 (SARS-CoV-2) and the subsequent Coronavirus Disease-19 (COVID-19), all sports in Norway suddenly stopped in mid-March 2020. Consequently, the 2020 Norwegian male premier league that was scheduled to start on the 4th of April was postponed until the 16th of June, thus shortening the fixture calendar by 49 days.

To fully recover from football matches, players need a recovery period of up to 72 h (1, 2). Resuming match play before players are fully recovered may lead to them playing with decreased muscular function (3), muscle tissue damage (4) and mental fatigue (5). Previous research has shown that injury risk can be affected when matches are congested into shorter periods (6–10). Research examining match congestion effects on injury rates has used many different thresholds and definitions. Matches played with four or fewer recovery days had higher injury rates than matches with six or more recovery days (9). Injury rates also increased when matches were played in succession with 3 days of recovery (6), when teams had 5 days or less recovery (8) and when teams had 3 days or less recovery (7). Furthermore, Howle et al. (10) found an increase in injury rates in weeks with more than one match and in seasons containing periods of match congestion. Contrary to these findings, Carling et al. (11) found no difference in injury rates in periods of match congestion (eight matches in 26 days). The first study examining the effect of rescheduling the fixture calendar due to COVID-19 was the German Bundesliga observing an increase in match injury incidence following a lockdown period compared to the pre-lockdown match period (12). To decrease individual match load, Federatione Nationale de Futbol (FIFA) introduced a rule change allowing teams to use five substitute players per match (13).

It is unknown how the changed game schedule affected injuries in professional leagues not used to match congestion. To inform federations and league organizations, technical and medical staff in future planning of match and training schedules, an investigation of the effect of the more match congested 2020 season on injury rates is warranted. Therefore, this study aimed to investigate seasonal differences in injury characteristics between the 2019 and 2020 seasons.

## Materials and methods

This longitudinal descriptive study compared the injury characteristics in two seasons in the Norwegian male football premier league (Eliteserien). All teams that participated in both the 2019 and 2020 (*N* = 13) seasons were invited. We included teams that, in their club setting, had prospectively recorded injuries in an electronic medical journal system, using the same medical staff to register injuries in both seasons. Toward the end of the 2020-season, we contacted each team's medical coordinator to introduce them to the study and inquire about their injury registration routines. All players with a first-team contract in 2019 and/or 2020 were invited. The study was reviewed by the Norwegian School of Sport Sciences' Ethical committee and approved by the Norwegian Centre for Research Data (896416). All eligible players signed written informed consent before the study start. We prepared this study according to the International Olympic Committee (IOC) consensus statement on methods for recording and reporting on epidemiology data in sport, and the STROBE Extension for Sports Injury and Illness Surveillance (14).

### Data collection

All data were anonymized by the team's responsible medical staff member and exported to the principal investigator (TDL) *via* email or post. Six teams manually extracted injury data from the medical records and/or their data records, whereas two teams exported data directly from their Athlete Monitoring System (AMS). When organizing the data into comparable spreadsheets, the principal investigator had video or telephone consultations with each of the teams' medical coordinators to ensure that all data were comparable across the two seasons and to exclude any data recording errors.

### Injury

Before the study start, we defined that only time-loss injuries would be included (14, 15). We ensured that all team's had used the same interpretation. All reported that they used the same criteria for return to play, i.e., when a player was cleared for full participation in either team training or match play. The number of days injured starting from the day after the onset of the injury (i.e., the first potential absence from team training activity) until the return to full participation was considered days lost to injury and used to calculate injury burden. When analyzing the injury burden, all days lost to injury were assigned to the month the injury was registered (i.e., an ACL injury happening in January 2019, would be attributed 300 days lost to injury and 30 matches missed to the January statistics). Injury severity was calculated based on the number of days lost per injury and categorized as recommended in the IOC consensus statement (14). Availability was calculated as the average percentage of players available for match selection. If a player was absent due to a reason other than an injury, the player was removed from the available player's calculation. The absence of players was expressed using the average percentage of players that were absent from training or match due to injury or illness.

### Exposure

We used data from the Football Association of Norway to record each teams' match exposure. All match exposures were calculated as *11 players X 90 minutes – minutes missed from red cards*, and we included league matches for the match exposure analysis. Since only three teams reported training exposure data, we excluded this data from the analyses.

### Data analyses

Continuous data are presented as mean (standard deviation; SD). Incidence was calculated in R (16) using the *epiR*-package (17) (script and data available as Supplementary Data 1). Incidence was expressed as the number of injuries per 1,000 h of exposure. Injury burden was expressed as the sum of all days off caused by injury.

When analyzing between season-difference in incidence and the number of injuries, a Poisson regression was used. The analysis was performed in R using the *sandwich* (18) and *msm* (19) packages (script and data available as Supplementary Data 2) and was reported with robust standard errors (20). To analyze the difference in the number of days lost and matches missed due to injury, a one-sample *t*-test was used for the average of the team's seasonal difference in Stata (V.15.3- StataCorp LLC, College Station, Texas, USA) using the *t*-test-command (script and results available as Supplementary Data 3). We did not analyze monthly seasonal differences on either injury parameter, as we considered the data insufficient for more detailed exploration.

## Results

We recorded 3,461 and 3,462 h of match exposure from the 2019 and 2020 seasons, respectively. A total of 505 injuries were recorded (Table 1), of which 183 occurred during match play. In total, we found 13,963 days lost and 1,370 matches missed due to injury.

**Table 1:** Overview of recovery days between matches in the two seasons.

**Days between matches**	***N*** **of games**	
	**2019**	**2020**
2	1	6
3	1	6
4	1	0
5	1	1
6	17	10
7	2	2
8	1	0
9	0	0
10	0	0
11	0	0
12	0	1
13	3	3

Games with less than four recovery days are highlighted.

### Participants and exposure

**Figure 1:**
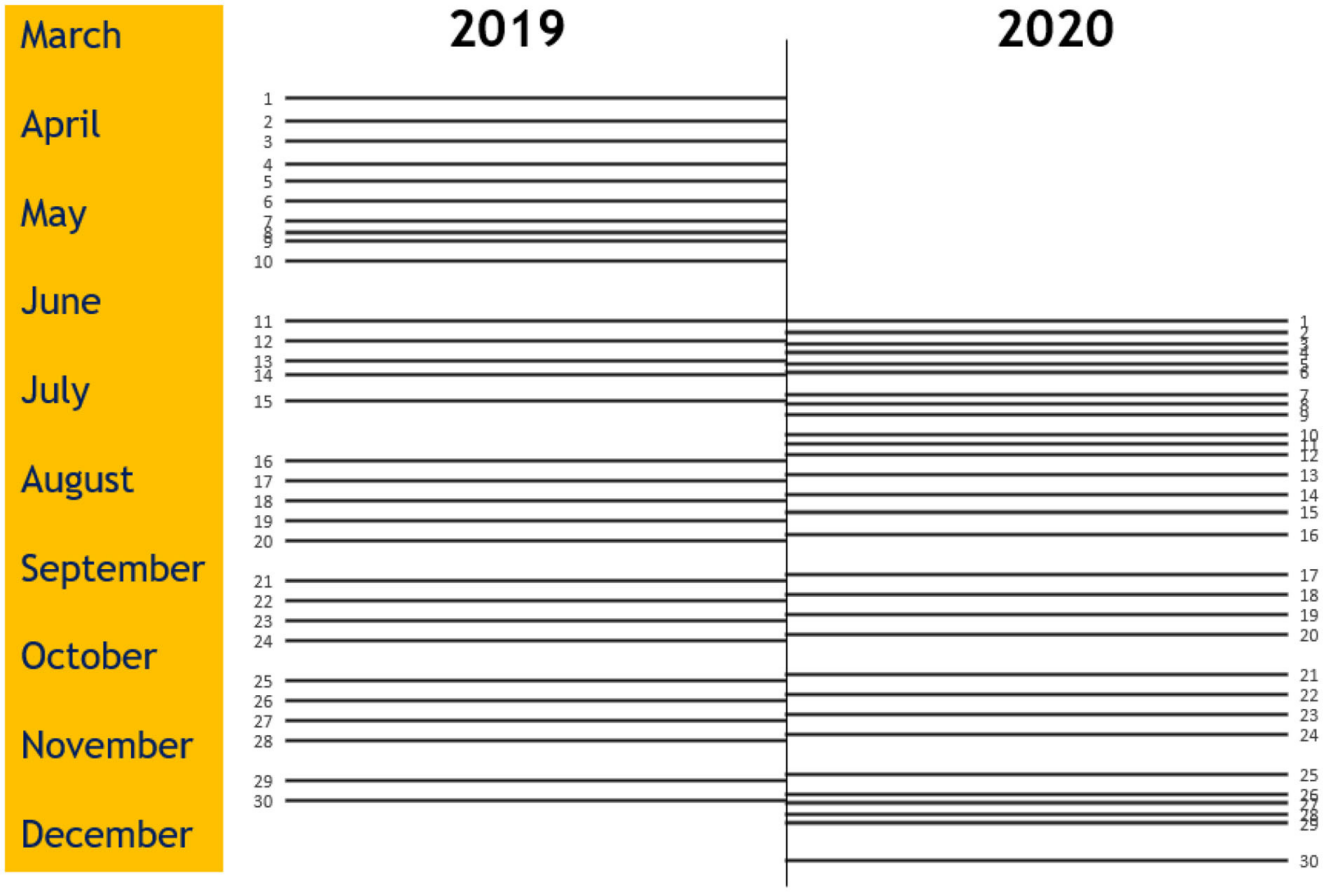
Distribution of matches in the Norwegian male premier league in the 2019 and 2020 seasons. One line represents the main match day for each round. The number represents the game week number.

The 2019-season started on the 31st of March and ended on the 1st of December, lasting 246 days. Due to COVID-19 restrictions, the 2020-season was postponed from the scheduled start on the 5th of April until the 16th of June and ended on the 22nd of December, reducing the planned match period from 238 to 189 days (Figure 1). The average number of recovery days between matches was 7.5 and 5.5 days in 2019 and 2020, respectively. However, the number of recovery days differed vastly between periods within both seasons, especially in game weeks 1 to 12 and 25 to 29 in 2020 (Figure 1; Table 1).

**Figure 2:**
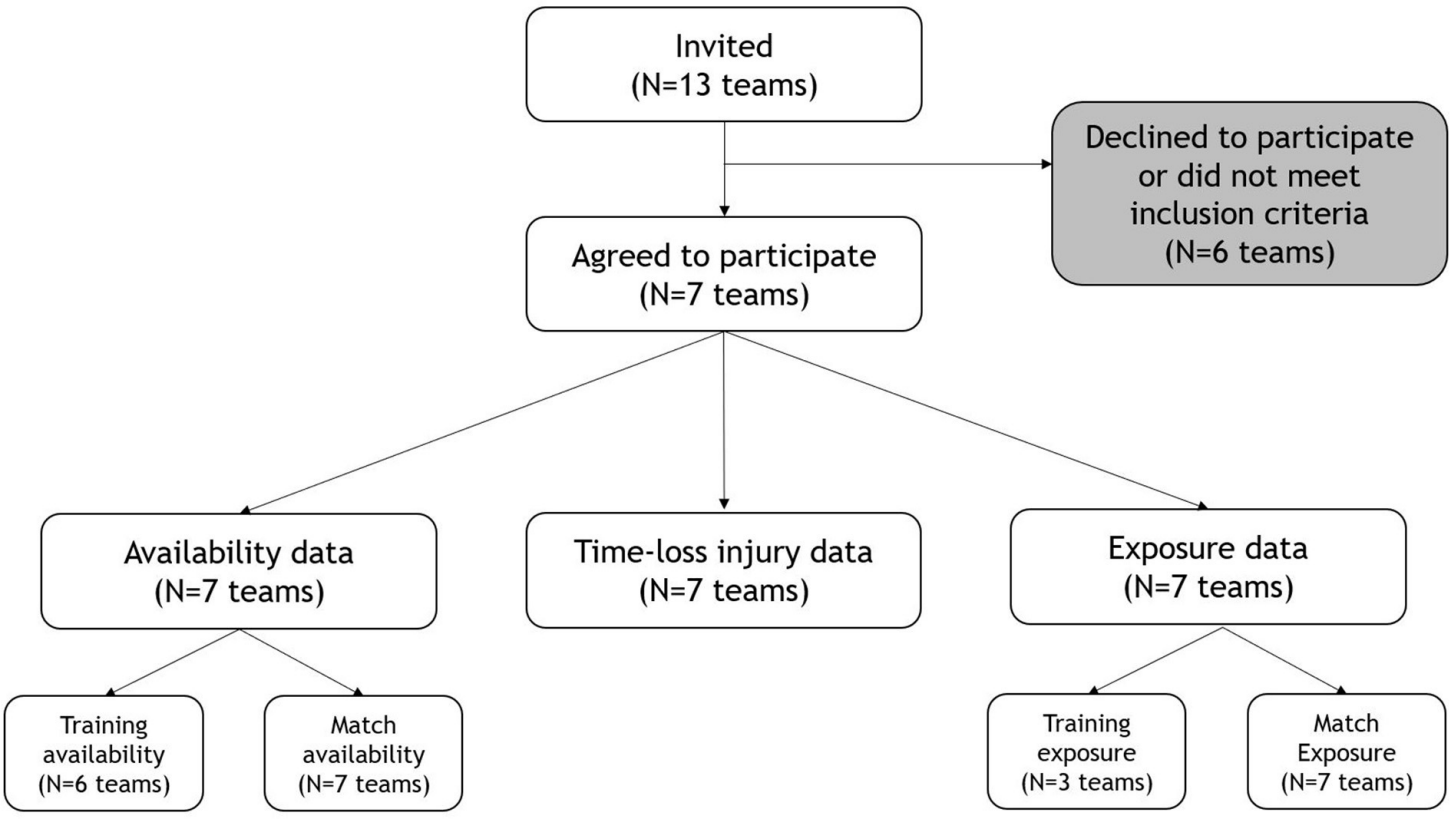
Flowchart of teams invited to participate in the study and the information obtained from the teams that were included. Thirteen teams were eligible as they were part of both the 2019 and 2020 campaigns.

Seven of 13 eligible teams agreed to participate (Figure 2). These teams had on average 26 players in their squad, and we included 213 players in the 2019 season and 208 in the 2020 season, giving a total of 421 player seasons. Of the six teams that were not included in the study, three teams had not registered injury data appropriately. One team had changed athlete management system (AMS) and felt they did not have comparable data. One team reported they did not have the resources to organize and export the data and one team declined without providing any reason (Figure 2).

### Injury incidence

The number of all injuries ranged from one team reporting seven injuries in the 2019 season to another team reporting 87 injuries in the 2020 season. There were in total 13 more injuries recorded in the 2020 season than in the 2019 season (1.05, Confidence Interval; CI 0.54–2.04; *p* = 0.88; Table 1).

### Match injury incidence

**Figure 3:**
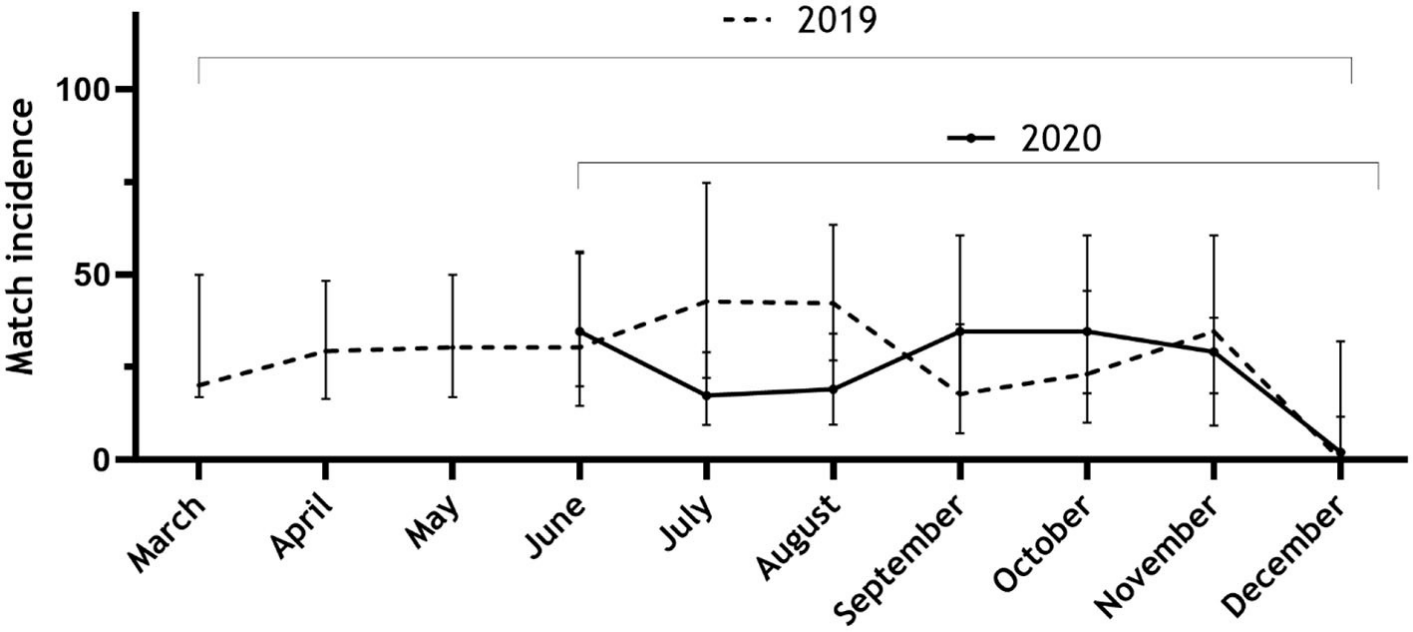
Timeline of monthly match injury incidence across the 2019 and the 2020 seasons.

We recorded 104 match injuries in the 2019 season and 79 match injuries in the 2020 season (Table 1). There was a large between-team discrepancy in match injury incidence (Table 2), ranging from 4.04 per 1,000 h to 48.54 per 1,000 h. The total match incidence was 7.23 per 1,000 h lower in 2020 (22.82 per 1,000 h; CI 18.07–28.44; Incidence Rate Ratio; IRR 0.76) than in 2019 (30.05 per 1,000 h; CI 24.55–36.41), however, this was not a significant difference (Table 2). The match incidence did not appear to follow a distinct pattern in either of the seasons (Figure 3).

**Table 2:** Number of match injuries, all injuries, total days lost due to injury and matches missed for the 2019 and the 2020 seasons.

**Team**	**Match injuries**			**All injuries**			**Total days lost**			**Matches missed**		
	**2019**	**2020**	**Change**	**2019**	**2020**	**Change**	**2019**	**2020**	**Change**	**2019**	**2020**	**Change**
1	4	2	−2	7	9	2	387	362	−25	40	47	7
2	18	10	−8	63	87	24	752	995	243	70	100	30
3	24	17	−7	71	44	−27	2,300	1,745	−555	204	126	−78
4	21	17	−4	38	44	6	771	1,037	266	95	116	21
5	11	15	4	18	30	12	516	540	24	46	72	26
6	15	8	−7	30	24	−6	1,205	1,057	−148	116	105	−11
7	11	10	−1	19	21	2	1,105	1,191	86	99	134	35
X / Sum	104	79	−25	246	259	13	7,036	6,927	−109	670	700	30

### Injury burden

**Figure 4:**
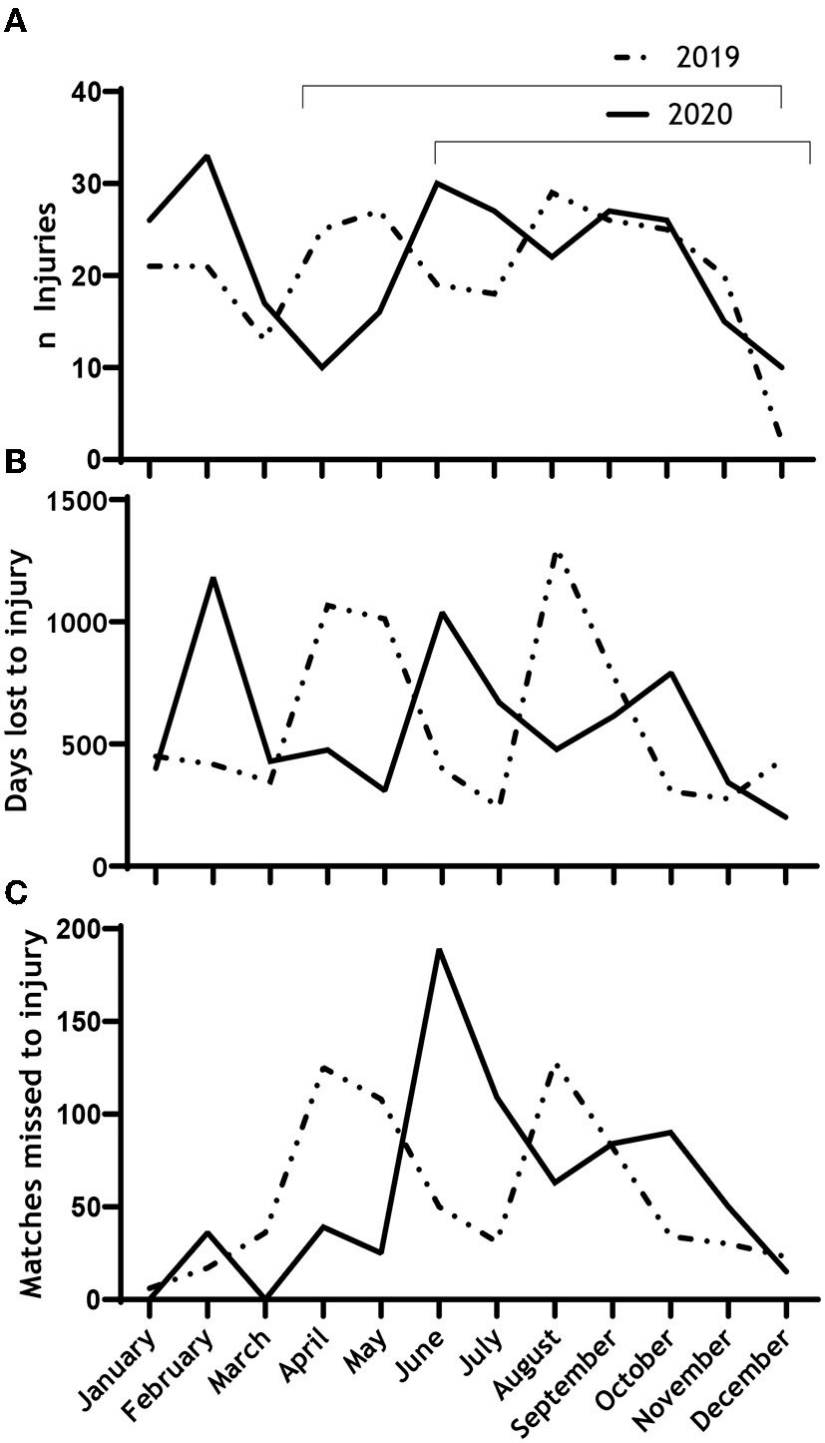
Timeline of the monthly number of injuries **(A)**, days lost due to injury **(B)** and matches lost due to injury **(C)** across the two seasons, 2019 and 2020.

We found no difference in the number of injuries (0.94, CI −0.49 to 1.8; *p* = 0.84), days lost to injury [−15.57 (CI −273.49 to 242.35); *p* = 0.89], or matches missed [4.28 (CI −32.26 to 40.83); *p* = 0.78] between the two seasons (Table 1; Figure 4).

### Availability

**Figure 5:**
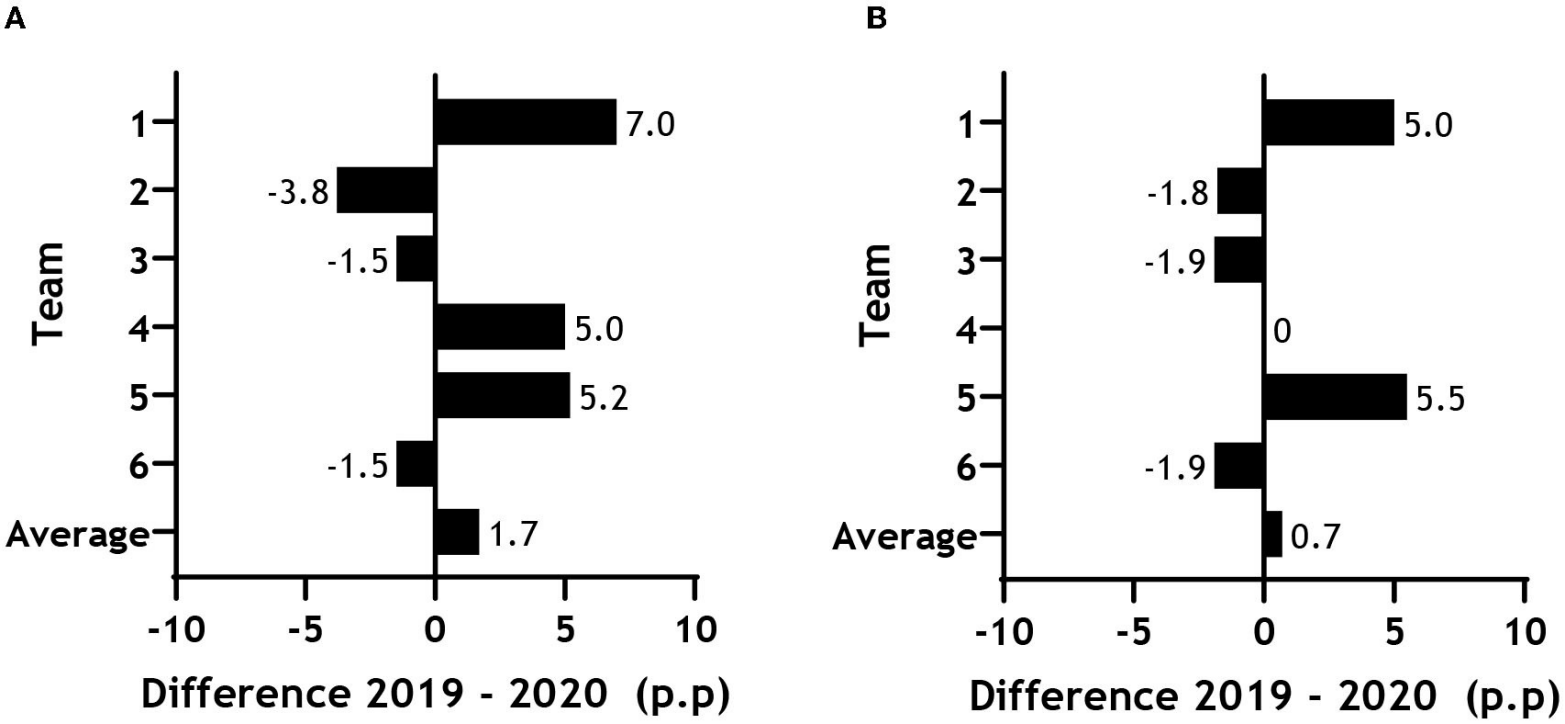
Training **(A)** and match **(B)** availability for all teams on average. Presented as percentage points difference from 2019 to 2020.

The average training availability was 84.1 and 85.9% in the 2019 and the 2020 seasons, respectively. The average match availability was 86.3% in the 2019 season and 87.1% in the 2020 seasons. Thus, we found no significant differences between the two seasons for neither training nor match availability (Figure 5).

### Injury severity

In the 2020-season, there were slightly fewer days lost to injury (*n* = 6,995 – *n* = 6,881). The injury severity seems to follow a similar distribution in both seasons, ~1/3 of the number of injuries are distributed in each of the categories mild, moderate and severe (Tables 3, 4).

**Table 3:** Match injury incidence in the 2019 and the 2020 seasons.

**Team**	**Match injury incidence**				
	**2019**	**2020**	**Change**	**Incidence rate ratio**	***p*** **-value**
1	8.09 (2.2–20.72)	4.04 (0.49–14.61)	−4.05		
2	36.36 (21.55–57.47)	20.21 (9.69–37.16)	−16.16		
3	48.54 (31.01–72.22)	34.34 (20.01–54.99)	−14.20		
4	42.42 (26.26–64.85)	34.43 (20.06–55.13)	−7.99		
5	22.29 (11.13–39.89)	30.39 (17–50.12)	8.10		
6	30.39 (17–50.12)	16.16 (6.97–31.84)	−14.23		
7	22.22 (11.09–39.76)	20.22 (9.69–37.18)	−2.00		
Average	30.05 (24.55–36.41)	22.82 (18.07–28.44)	−7.23	0.76 (0.48–1.20)	0.24

Average difference highlighted.

**Table 4:** Number of injuries and total days lost due to injury categorized by their severity.

	**Number of injuries**			**Total days lost to injury**		
**Category (days)**	**2019**	**2020**	**Diff**	**2019**	**2020**	**Diff**
Slight (0)	0	1	1	0	0	0
Mild (1–7)	90	96	6	313	295	−18
Moderate (8–27)	87	93	6	1,294	1,449	155
Severe (>28)	69	70	1	5,388	5,137	−251
All	246	260	14	6,995	6,881	−114

## Discussion

This study aimed to compare potential differences in the injury patterns in one regular season and one more congested season in Norwegian male professional football. The planned congested match schedule raised concerns among players and coaches related to match load and injury risk. Based on our data from seven out of the 13 teams, there was no increase in injuries in 2020 and the players' and coaches' concerns were unjustified.

### Match injury incidence

The match injury incidence was higher compared to previous studies from the Norwegian male premier league (21). Bjørneboe et al. found an overall increase in match injury incidence from 2002 to 2007, the increase found then is likely to have continued (21). The match injury incidence in this study is comparable with findings from the comprehensive UEFA Champions League injury audit (23 per 1,000 h) (22). Our results (30 per 1,000 h and 23 per 1,000 h, respectively) are slightly lower than the match injury incidence of 36 per 1,000 h reported by López-Valenciano et al. (23) in a meta-analysis of 40 studies in professional male football.

We did not observe a between-season difference, even though the 2020-season was played with an average of two fewer recovery days between matches and ten more games played with 2 or 3 days between matches (Table 1). Previous studies examining the effect of a congested match schedule have reported increased (6–11) and unchanged (24, 25) injury rates. Most of these studies have investigated whether shorter periods of match congestion lead to increased injury risk compared with match periods with more recovery days in between (6–9, 11, 24, 25). We compared two complete seasons with very different match scheduling (Figure 1; Table 1), making a direct comparison with most previous studies difficult. One exemption, however, is the study by Howle et al. (10) who compared three full seasons and found that the season with congested match periods had higher injury rates. This was not in line with our results. Despite the 2020 season having 5.5 recovery days on average, many match periods were even more congested (Figure 1; Table 1). For instance, following a positive COVID-19 test in one team, the team was quarantined for 10 days and not allowed any scheduled team training. Immediately following the quarantine period, the team played five matches in 13 days, resulting in three match injuries.

Four studies have used public databases for investigating differences in injuries after the COVID-19 lockdown (12, 26–28). Of these, only Marotta et al. (28) have collected full season data. The other studies have either compared pre- and post-lockdown data alone (12, 28), or compared to several comparison periods (27).

Seshadri et al. (12) reported a 3-fold increase in injury rate when the German Bundesliga resumed playing after 2 months in lockdown. Krutsch et al. (27) compared the post lockdown German Bundesliga with three comparison periods. They found no difference post lockdown compared to the period directly before, compared to the start of the season, but lower incidence than the same period in the previous season. Marotta et al. (28) investigated match injuries in the Italian Serie A before and after lockdown and found no differences. Mannino et al. (26) reported an increased injury incidence in the 2020–2021 premier league season compared to the 2018–2019 and 2019–2020 seasons. The preparation period before resuming match play post lockdown was 4 weeks in the Serie A and only 10 days in the Bundesliga (28, 29), different to the 12 weeks in the Norwegian League. Two papers have used data prospectively collected in the teams. Moreno-Pérez et al. (30) compared the 11 games played after the lockdown to the first 27 games of the season and found no difference in match injury incidence. Waldèn et al. (31) used injury data from the resumption of competition of twelve European top-level teams and compared with the data from corresponding periods from 2015 to 2019. They reported no difference in match injury incidence, which is similar to our findings and the majority of other studies (26, 28, 30).

Due to the pandemic, the Norwegian male premier league preseason was postponed for 2 months (mid-March), then players being allowed to train in small cohorts of five from mid-April to mid-May. After mid-May, normal-proximity team training and friendly matches were allowed for 4 weeks before the season started in mid-June. The 4 week period of regular preseason preparation in the Norwegian premier league is likely to have mitigated some of the injury risk (32).

In an attempt to decrease the individual match load on the players', teams were permitted five substitutions in the 2020-season, compared to three in the 2019-season (13). This affected the number of substitutions, as the average number of substitutions increased by 0.8 per match (2.8 in 2019, 3.6 in 2020) in the teams participating in this study. Moreover, this rule change has enabled teams to rotate players and manage the load of players individually based on perceived risk of injury, possibly contributing to mitigating some of the injury risk in the 2020-season.

### Availability, injury severity and injury burden

Periods of match congestion can lead to a decrease in weekly training load (33), and thus expose players to injury during training affecting the overall number of injuries. We recorded an average training (84 and 86%, respectively) and match availability (86 and 87%, respectively), similar to the previously reported training availability (88%) and match availability (88%) by Ekstrand et al. (22) We did not find any differences in days lost to injury between the two seasons. In periods of match congestion, the same number of days lost to injury would mean more matches missed than in a normal period (i.e., a 2-week absence in mid-June would result in zero matches missed in 2019 and five matches missed in 2020). This was not the case in our study, nor regarding matches missed due to injury or match availability.

### Methodological considerations

What constitutes a “recordable event” is arguably one of the most critical methodological factors in sports injury and illness surveillance studies (34). In this study, we used data from the teams' injury surveillance systems, and differences in perception of what constitutes a recordable event could explain the large inter-team variation. Surveillance data from different data recorders are not necessarily comparable (35), and therefore, we only compared each team's data with their own data. We chose to use a time-loss definition as it is considered the most reliable definition, because full participation in training or match play is relatively easy to measure, and is considered reliable across recorders (36). There are, however, a large number of injury problems that do not lead to reduced participation, which are overlooked using the time-loss definition (34, 37, 38).

One major limitation in this study is the lack of training exposure data. This was not made accessible by the teams, and therefore, we were prevented from calculating the recommended metrics of the overall incidence and injury burden per 1,000 h in this study (14, 39). Furthermore, only using one season as a baseline for what is “normal” is a limitation injury rates will vary from season to season (24, 40). Hence, we cannot be sure that the 2019-season is a correct measure of a regular Norwegian premier league season. Still, compared to studies examining only periods of the season, our full-season design does not have any bias caused by differences in opponents. Also, our data were prospectively collected by the club's medical staff and not from public databases, which we consider a strength of our design. Furthermore, as we only compared data on the two full seasons, we do not have any specific data on the periods that were congested. Some parts of the 2020-season were not affected by congestion at all (Figure 1) and will affect the season-long average. On the other hand, when reviewing Figures 3, 4, there is no clear trend of injuries and congestion.

Our findings are not necessarily comparable to the top-elite leagues in Europe. A regular competitive season in the Norwegian premier league involves an average of ~4.6 matches per month from April to November. This is a lower number of monthly matches compared to top-level teams in international leagues who play approximately six matches per month for 10 months (7). However, these findings may inform practice for leagues having similar schedules as the Norwegian league.

### Perspectives

Despite the limitations of this study, our results can inform federations and league organizations in scheduling competitive season setup. The rule change implemented due to the pandemic which allowed five substitutions per match enabled teams to incorporate and improve rotation strategies. This could be one of several potential reasons that may have mitigated an increased injury risk due to match congestion. We think this should be considered when planning seasons with unexpected or unusual high match congestion in the future. Our findings are especially applicable for leagues playing a similar amount and frequency of matches.

### Practical implications

Based on the data from this study, playing a more match congested calendar congestion may be safe when using safety measures such as an allowance of increased substitutions. At the same time, we cannot exclude that congested periods might be harmful as we only investigated season averages.

## Data availability statement

The raw data supporting the conclusions of this article will be made available by the authors, without undue reservation.

## Ethics statement

Ethical review and approval was not required for the study on human participants in accordance with the local legislation and institutional requirements. The patients/participants provided their written informed consent to participate in this study.

## Author contributions

All authors listed have made a substantial, direct, and intellectual contribution to the work and approved it for publication.

## Conflict of interest

Author CT was employed by the company Rosenborg Ballklubb. Author MB was employed by the company Bodø Glimt FK. Author DT was employed by the company Brann Sportsklubb. Author AB was employed by the company Odds Ballklubb. Author CW was employed by the company Kristiansund Ballklubb. Author EM was employed by the company Stabæk Fotball. The remaining authors declare that the research was conducted in the absence of any commercial or financial relationships that could be construed as a potential conflict of interest.

## Publisher's note

All claims expressed in this article are solely those of the authors and do not necessarily represent those of their affiliated organizations, or those of the publisher, the editors and the reviewers. Any product that may be evaluated in this article, or claim that may be made by its manufacturer, is not guaranteed or endorsed by the publisher.
